# A cross-sectional study to test equivalence of low- versus intermediate-flip angle dynamic susceptibility contrast MRI measures of relative cerebral blood volume in patients with high-grade gliomas at 1.5 Tesla field strength

**DOI:** 10.3389/fonc.2023.1156843

**Published:** 2023-09-20

**Authors:** Mark S. Shiroishi, Dane Weinert, Steven Y. Cen, Bino Varghese, Timothy Dondlinger, Melissa Prah, Jesse Mendoza, Sina Nazemi, Nima Ameli, Negin Amini, Salman Shohas, Shannon Chen, Bavrina Bigjahan, Gabriel Zada, Thomas Chen, Josh Neman-Ebrahim, Eric L. Chang, Frances E. Chow, Zhaoyang Fan, Wensha Yang, Frank J. Attenello, Jason Ye, Paul E. Kim, Vishal N. Patel, Alexander Lerner, Jay Acharya, Leland S. Hu, C. Chad Quarles, Jerrold L. Boxerman, Ona Wu, Kathleen M. Schmainda

**Affiliations:** ^1^ Department of Radiology, Keck School of Medicine of the University of Southern California (USC), Los Angeles, CA, United States; ^2^ Imaging Genetics Center, USC Mark and Mary Stevens Neuroimaging and Informatics Institute, Marina del Rey, CA, United States; ^3^ Department of Population and Public Health Sciences, Keck School of Medicine of USC, Los Angeles, CA, United States; ^4^ Imaging Biometrics, Elm Grove, WI, United States; ^5^ Department of Biophysics, Medical College of Wisconsin, Milwaukee, WI, United States; ^6^ Department of Neurological Surgery, Keck School of Medicine of USC, Los Angeles, CA, United States; ^7^ Department of Radiation Oncology, Keck School of Medicine of USC, Los Angeles, CA, United States; ^8^ Department of Radiology, Mayo Clinic, Jacksonville, FL, United States; ^9^ Department of Radiology, Mayo Clinic, Phoenix, AZ, United States; ^10^ Cancer Systems Imaging, The University of Texas MD Anderson Cancer Center, Houston, TX, United States; ^11^ Department of Diagnostic Imaging, The Warren Alpert Medical School of Brown University, Providence, RI, United States; ^12^ Athinoula A. Martinos Center for Biomedical Imaging, Massachusetts General Hospital, Harvard Medical School, Boston, MA, United States

**Keywords:** dynamic susceptibility contrast MRI (DSC-MRI), flip angle, standardization, glioma, 1.5 Tesla

## Abstract

**Introduction:**

1.5 Tesla (1.5T) remain a significant field strength for brain imaging worldwide. Recent computer simulations and clinical studies at 3T MRI have suggested that dynamic susceptibility contrast (DSC) MRI using a 30° flip angle (“low-FA”) with model-based leakage correction and no gadolinium-based contrast agent (GBCA) preload provides equivalent relative cerebral blood volume (rCBV) measurements to the reference-standard acquisition using a single-dose GBCA preload with a 60° flip angle (“intermediate-FA”) and model-based leakage correction. However, it remains unclear whether this holds true at 1.5T. The purpose of this study was to test this at 1.5T in human high-grade glioma (HGG) patients.

**Methods:**

This was a single-institution cross-sectional study of patients who had undergone 1.5T MRI for HGG. DSC-MRI consisted of gradient-echo echo-planar imaging (GRE-EPI) with a low-FA without preload (30°/P-); this then subsequently served as a preload for the standard intermediate-FA acquisition (60°/P+). Both normalized (nrCBV) and standardized relative cerebral blood volumes (srCBV) were calculated using model-based leakage correction (C+) with IBNeuro™ software. Whole-enhancing lesion mean and median nrCBV and srCBV from the low- and intermediate-FA methods were compared using the Pearson’s, Spearman’s and intraclass correlation coefficients (ICC).

**Results:**

Twenty-three HGG patients composing a total of 31 scans were analyzed. The Pearson and Spearman correlations and ICCs between the 30°/P-/C+ and 60°/P+/C+ acquisitions demonstrated high correlations for both mean and median nrCBV and srCBV.

**Conclusion:**

Our study provides preliminary evidence that for HGG patients at 1.5T MRI, a low FA, no preload DSC-MRI acquisition can be an appealing alternative to the reference standard higher FA acquisition that utilizes a preload.

## Introduction

Dynamic susceptibility contrast (DSC)-MRI-derived relative cerebral blood volume (rCBV) is the most commonly used brain tumor perfusion imaging metric. Given inherent limitations of conventional contrast-enhanced MRI, DSC-MRI may provide valuable insight into important questions such as therapeutic response and overall-survival in brain tumor patients, particularly those with high-grade glioma (HGG) (1–6). Despite being developed more than 3 decades ago (7, 8), widespread adoption of DSC-MRI remains limited, largely stemming from a lack of a consensus on the optimal methods of imaging data acquisition and analysis as well as user perceptions of complex methodology. Recent computer simulations (9, 10) and clinical studies (11) of HGG patients at 3T MRI have suggested that a low-flip-angle (FA) (30°) DSC-MRI acquisition with model-based leakage correction and no GBCA preload provide equivalent rCBV measurements to the reference-standard acquisition method consisting of single-dose gadolinium-based contrast agent (GBCA) preload with intermediate-FA (60°) and model-based leakage correction (10–12). If confirmed in larger multicenter trials, it could promote more widespread use of DSC-MRI given the more simplified acquisition of a single, rather than double dose, administration of a GBCA. While these results pertain to 3T MRI scanners, 1.5T likely still provides the bulk of brain scanning around the world (13); the major scanner manufacturers still currently produce new 1.5T scanners (13–16). Therefore, for HGG patients at 1.5T, it is critical to know whether this no preload, low-FA approach may also be a suitable substitute for the reference-standard intermediate-FA approach with preload. The purpose of this study was to address this question.

## Materials and methods

### Patients

This single institution cross-sectional study was performed at the Los Angeles County + University of Southern California (LAC+USC) Medical Center. This study received Institutional Review Board approval (IRB #HS-20-00859) with informed consent waived for this retrospective study. Inclusion criteria were as follows: 1. Adult HGG patients who underwent conventional and DSC-MRI with both low- and intermediate-FAs at 1.5T MRI. 2. The presence of a contrast-enhancing lesion at least 10 mm in size. 3. Scans could be obtained before or after treatment. Exclusion criteria were as follows: Scans that had major artifacts stemming from susceptibility artifacts, motion artifacts, poor GBCA bolus injection and other miscellaneous technical problems patients were scanned between 2020-2022.

### Imaging

**Figure 1:**
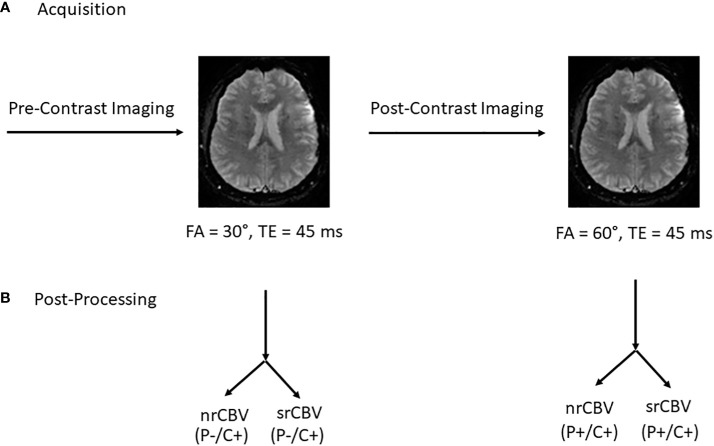
Acquisition protocol and post processing overview. Following standard pre-contrast imaging sequences, low-(30°) flip angle (FA) DSC-MRI was performed (gradient recalled-echo EPI, TR/TE (1500 ms/45 ms) without GBCA preload (P) using a GBCA bolus injection of 0.1 mmol/kg of gadoterate meglumine. Subsequently postcontrast T1-weighted images were obtained and a second DSC-MRI was acquisition was performed using an intermediate FA (60°) and otherwise identical acquisition parameters and GBCA dose. The time between the first and second DSC-MR imaging study was about 8 minutes. The low-FA DSC-MRI acquisition function as the GBCA preload for the subsequent intermediate-FA DSC-MRI acquisition **(A)**. Post-processing involved the calculation of both nrCBV and srCBV for both low- and intermediate-FA DSC MRI acquisitions **(B)**. P = GBCA preload, C = model-based leakage correction.

All scans were performed on one of three 1.5T GE Signa Explorer Lift scanners, software version 25.0. **Figure 1** depicts the imaging acquisition and post-processing scheme. Following standard T1-weighted, T2-weighted and T2-weighted FLAIR imaging, low-(30°) FA DSC-MRI was performed (gradient recalled-echo EPI, TR/TE (1500 ms/45 ms) without GBCA preload using a GBCA bolus injection of 0.1 mmol/kg of gadoterate meglumine (Dotarem; Guerbet, Paris, France). Additional DSC-MRI scan parameters included the following: FOV = 220 mm, matrix 128 x 128, slice thickness 4 mm with no gap to cover the enhancing lesion and 60 seconds of pre-contrast baseline data (about 40 time points) with a total of 100 time points acquired. Subsequently postcontrast T1-weighted images were obtained and a second DSC-MRI acquisition was performed using an intermediate-FA (60°) and otherwise identical acquisition parameters and GBCA dose. The time between the first and second DSC-MR imaging study was about 8 minutes. The low-FA DSC-MRI acquisition served as the GBCA preload for the subsequent intermediate-flip angle DSC-MRI acquisition.

### Image analysis

**Figure 2:**
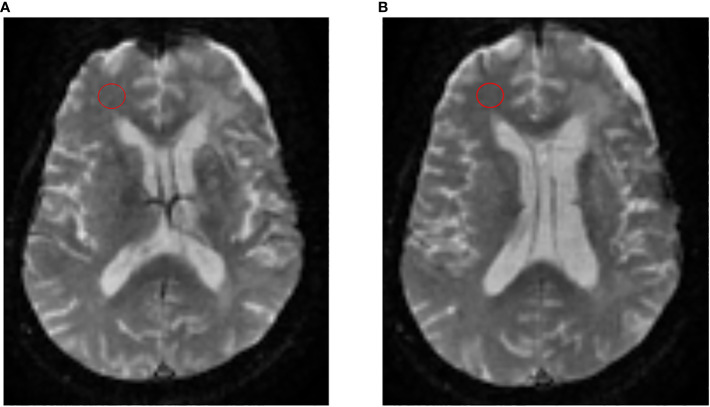
Process to create nrCBV maps. To create nrCBV maps, 2 circular 8 mm regions of interest (ROIs) were placed by a single user on consecutive axial slices **(A, B)** at the midventricular level near the frontal horns of the lateral ventricles in the contralateral normal appearing white matter (NAWM).

All scans were anonymized and post-processed using OsiriX Imaging Software (http://www.osirix-viewer.com) with IB Neuro™ and IB Delta Suite™ plugins (Imaging Biometrics, Elm Grove, Wisconsin) to create both normalized rCBV (nrCBV) and standardized rCBV (srCBV) maps (17). To create nrCBV maps, two circular 8 mm regions of interest (ROIs) were placed by a single user (DW – radiology resident with 2 years experience, supervised by an attending CAQ qualified, board certified academic neuroradiologist MSS – 15 years of experience) on consecutive axial slices at the midventricular level near the frontal horns of the lateral ventricles in the contralateral normal appearing white matter (NAWM) (**Figure 2**). These ROIs were placed in the NAWM of the contralateral occipital lobe if the region near the frontal horns of lateral ventricles was abnormal. The creation of the srCBV maps does not require the manual placement of an ROI in the contralateral NAWM. Rather, it utilizes a standardization approach where a machine-learned calibration rule results in quantitative rCBV maps that are consistent across scanners, time and patients (17–19).

### Statistical analysis

Given our relatively small sample size, neither histogram nor normality tests could appropriately adjudicate data normality. Therefore, we conducted both Pearson’s and Spearman’s correlation tests. If both tests agreed, then we can be confident of the correlation results. If they disagreed, Spearman’s correlation was used. For measurement agreement, we used intraclass correlation coefficient (ICC3:1:2-way mixed with absolute agreement), with corresponding 95% confidence intervals. ICC 2-way mixed (ICC3,1) has the same value as Lin’s concordance correlation. Comparisons were conducted for nrCBV and srCBV acquired at 30°/P-/C+ and 60°/P+/C+. Bland-Altman plots and scatter plots were used to illustrate the correlations, agreement and potential pattern of bias. Statistical analysis was performed using SAS 9.4 (Cary, NC, USA). Several patients in our sample contributed multiple follow-up scans. To address the concern that our results could have been biased due to the inclusion of these repeat scans, we conducted sensitivity analysis wherein we repeated our analysis by including only the very first scan for those subjects where follow-up scans were conducted. We also assessed for differences in associations whether the normalizing ROIs were placed in the contralateral NAWM near the frontal horns of the lateral ventricles or in the NAWM of the contralateral occipital lobe.

## Results

A total of 33 patients with 45 MRI scans were identified for analysis. However, 14 scans had to be excluded (8 - poor contrast agent bolus delivery, 3 - severe susceptibility artifact and 3 - motion artifacts). Ultimately, a total of 23 subjects, some of whom had follow-up scanning, comprised 31 total MRI scans that satisfied all the inclusion and exclusion criteria. Twenty-eight were GBM scans while 3 were Grade III anaplastic astrocytomas. Further patient characteristics of these patients is given in **Table 1**.

**Table 1:** Patient characteristics.

Characteristic	(n=31)
Sex	
Male	24 (77.4%)
Female	7 (22.6%)
Age, mean (range)	53 (37-73)
Diagnosis	
Glioblastoma, WHO grade IV	28 (84.8%)
Anaplastic Astrocytoma, WHO grade III	3 (9.7%)
IDH status	
IDH1 wildtype	15 (48.4%)
IDH1 mutant	3 (9.7%)
Unknown	13 (41.9%)
MGMT promoter	
Methylated	6 (19.4%)
Unknown	25 (80.6%)
Number of recurrences	
Newly diagnosed	17 (54.8%)
1^st^ recurrence	9 (29.0%)
2^nd^ recurrence	9 (29.0%)
3^rd^ recurrence	2 (6.5%)
Treatment History	
Radiation	21 (67.7%)
Temozolomide	21 (67.7%)
Lomustine	7 (22.6%)
Bevacizumab	1 (3.2%)
Tumor Treating Fields	2 (6.5%)

**Figure 3:**
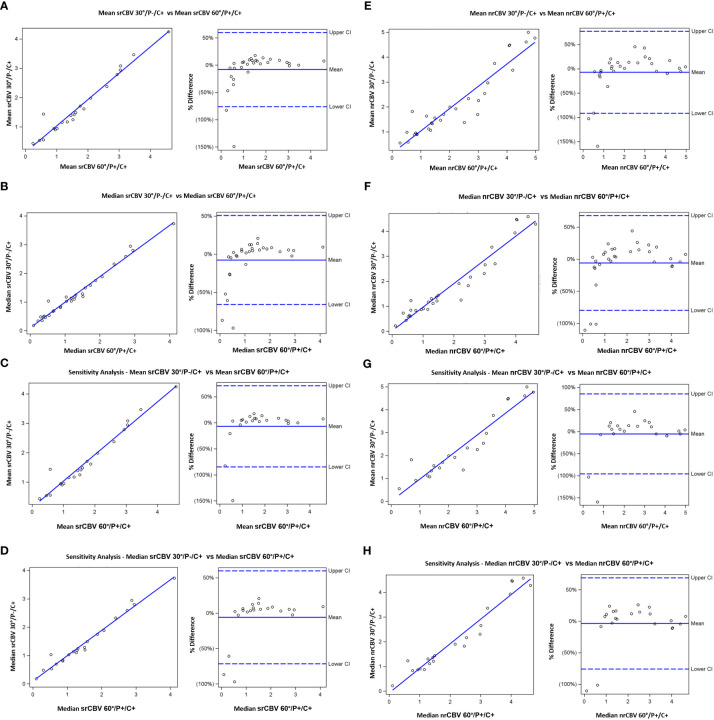
Scatterplots (left) showing results from Pearson correlation and (right) Bland-Altman plots of bias. Results shown are for mean srCBV **(A)**, median srCBV **(B)**, mean nrCBV **(C)**, median nrCBV **(D)** for the entire sample as well as for sensitivity analysis **(E–H)**.

**Figure 4:**
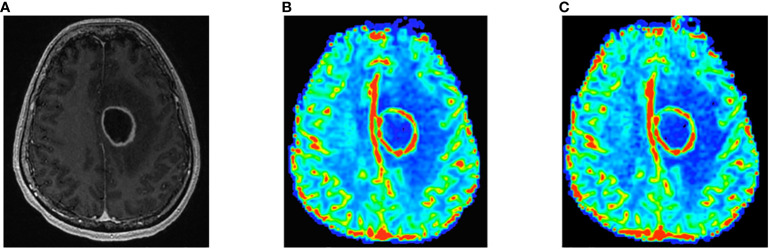
Contrast-enhanced T1-weighted image shows left frontal rim-enhancing mass consistent with GBM **(A)**. Note similarity of 60°/P+/C+ **(B)** and 30°/P-/C+ **(C)** color srCBV maps.

Pearson and Spearman correlations and ICCs between the 30°/P-/C+ and 60°/P+/C+ acquisitions for the analysis for the larger sample including follow-up scans for srCBV and nrCBV showed high correlations as shown in **Tables 2A–D**. Sensitivity analysis (performed to address the concern that our results could have been biased due to the inclusion of repeat scans as described above) also showed high correlations between the same comparison groups (**Tables 2E–H**). **Figure 3** illustrates both scatter plots and Bland-Altman plots. In most cases, the values were consistent and well correlated between 30°/P-/C+ and 60°/P+/C+ acquisitions. However, there were a few outliers where when the rCBV value was low (mean or median <1), the 30°/P-/C+ acquisition resulted in a higher rCBV than 60°/P+/C+. **Figure 4** demonstrates an example of the similarity of 30°/P-/C+ and 60°/P+/C+ srCBV color maps.

**Table 2:** Strong correlations were seen for the entire sample between mean and median srCBV 30°/P-/C+ vs srCBV 60°/P+/C+ (A, B) and mean and median nrCBV 30°/P-/C+ vs n rCBV 60°/P+/C+ (C, D).

Table 2A - Mean srCBV 30°/P-/C+ vs srCBV 60°/P+/C+			
Pearson r	Spearman r	ICC	Number of scans
0.98, 95% CI (0.96, 0.99)	0.95, 95% CI (0.90, 0.98)	0.98, 95% CI (0.95, 0.99)	31
Table 2B – Median srCBV 30°/P-/C+ vs srCBV 60°/P+/C+			
Pearson r	Spearman r	ICC	Number of scans
0.99, 95% CI (0.98, 0.99)	0.98, 95% CI, (0.96, 0.99)	0.98, 95% CI, (0.97, 0.99)	31
Table 2C - Mean nrCBV 30°/P-/C+ vs nrCBV 60°/P+/C+			
Pearson r	Spearman r	ICC	Number of scans
0.94, 95% CI, (0.88, 0.97)	0.91, 95% CI, (0.83, 0.96)	0.94, 95% CI (0.88, 0.97)	31
Table 2D – Median nrCBV 30°/P-/C+ vs nrCBV 60°/P+/C+			
Pearson r	Spearman r	ICC	Number of scans
0.97, 95% CI (0.93, 0.98)	0.97, 95% CI (0.94, 0.98)	0.97, 95% CI (0.93, 0.98)	31
Table 2E – Sensitivity Analysis - Mean srCBV 30°/P-/C+ vs srCBV 60°/P+/C+			
Pearson r	Spearman r	ICC	Number of scans
0.98, 95% CI, (0.95, 0.99)	0.95, 95% CI (0.89, 0.98)	0.98, 95% CI (0.94, 0.99)	23
Table 2F – Sensitivity Analysis - Median srCBV 30°/P-/C+ vs srCBV 60°/P+/C+			
Pearson r	Spearman r	ICC	Number of scans
0.99, 95% CI (0.97, 0.99)	0.98, 95% CI (0.94, 0.99)	0.98, 95% CI (0.96, 0.99)	23
Table 2G - Sensitivity Analysis - Mean nrCBV 30°/P-/C+ vs nrCBV 60°/P+/C+			
Pearson r	Spearman r	ICC	Number of scans
0.94, 95% CI, (0.87, 0.98)	0.93, 95% CI (0.84, 0.97)	0.95, 95% CI (0.88, 0.98)	23
Table 2H – Sensitivity Analysis - Median nrCBV 30°/P-/C+ vs nrCBV 60°/P+/C+			
Pearson r	Spearman r	ICC	Number of scans
0.97, 95% CI (0.93, 0.99)	0.96, 95% CI (0.89, 0.98)	0.97, 95% CI (0.94, 0.99)	23

Sensitivity analyses for the same group comparisons (performed to address the concern that our results could have been biased due to the inclusion of repeat scans, i.e. only the very first scans were used for this analysis) are displayed in (E–H) and also exhibited strong correlations. ICC, intraclass correlation coefficient; CI, confidence interval.

Furthermore, **Table 3** shows that strong correlations were seen regardless of if normalizing ROIs were placed in the contralateral NAWM near the frontal horns of the lateral ventricles (A and B) or in the NAWM of the contralateral occipital lobe (C and D between mean and median nrCBV 30°/P-/C+ vs nCBV 60°/P+/C+.

**Table 3:** Strong correlations were seen regardless of if normalizing ROIs were placed in the NAWM near the frontal horns of the lateral ventricles (A, B) or in the NAWM of the contralateral occipital lobe (C, D) between mean and median nrCBV 30°/P-/C+ vs nCBV 60°/P+/C+.

Table 3A - Normalization ROI’s placed near the frontal horns of the lateral ventricles in the contralateral normal appearing white matter (NAWM) - Mean nrCBV 30°/P-/C+ vs nrCBV 60°/P+/C+			
Pearson r	Spearman r	ICC	Number of scans
0.96, 95% CI (0.89, 0.98)	0.87, 95% CI (0.68, 0.95)	0.96 95% CI (0.89, 0.98)	18
Table 3B - Normalization ROI’s placed near the frontal horns of the lateral ventricles in the contralateral normal appearing white matter (NAWM) - Median nrCBV 30°/P-/C+ vs nrCBV 60°/P+/C+			
Pearson r	Spearman r	ICC	Number of scans
0.97, 95% CI (0.93, 0.99)	0.96, 95% CI (0.9, 0.99)	0.98, 95% CI (0.94, 0.99)	18
Table 3C - Normalization ROI’s placed in the NAWM of the contralateral occipital lobe - Mean nrCBV 30°/P-/C+ vs nrCBV 60°/P+/C+			
Pearson r	Spearman r	ICC	Number of scans
0.92, 95% CI (0.76, 0.98)	0.98, 95% CI (0.94, 0.99)	0.93, 95% CI (0.78, 0.98)	13
Table 3D - Normalization ROI’s placed in the NAWM of the contralateral occipital lobe - Median nrCBV 30°/P-/C+ vs nrCBV60°/P+/C+			
Pearson r	Spearman r	ICC	Number of scans
0.96, 95% CI (0.86, 0.99)	0.96, 95% CI (0.86, 0.99)	0.96, 95% CI (0.88, 0.99)	13

ICC, intraclass correlation coefficient; CI, confidence interval.

## Discussion

Currently, the use of a single-dose GBCA preload with intermediate FA (60°) and model-based leakage correction (60°/P+/C+) has generally been considered the reference standard DSC-MRI acquisition methodology for brain tumors due to multiple factors including its utility, accuracy and precision (1–3, 5, 19–23). Recent theoretical work by Leu et al. (9) and Semmineh et al. (10, 24) and clinical validation from Schmainda et al. (11) have suggested that a low-FA, no preload DSC-MRI acquisition is concordant with the reference standard method at 3T. However, up until now, it has remained unknown whether this holds true at 1.5T in clinical HGG patients. This is important to know because 1.5T MRI scanners remain a significant field strength for brain imaging worldwide (14–16).

Our results at 1.5T showed excellent correlation and ICC for both srCBV and nrCBV between the low-FA, no preload DSC-MRI acquisition with model-based leakage correction (30°/P-/C+) and the current reference standard intermediate-FA (60°/P+/C+) method. There was also similar excellent agreement in our sensitivity analysis where we only included the first MRI scans of those subjects who had follow-up scans; this demonstrates that our results are robust and not subject to bias from those subjects that had more follow-up examinations compared to the others. There was also excellent agreement regardless of if the normalizing ROIs were placed in the contralateral NAWM near the frontal horns of the lateral ventricles or in the NAWM of the contralateral occipital lobe.

These findings suggest that, similar to studies at 3T, a single GBCA injection low-FA acquisition method with model-based leakage correction at 1.5T might be a feasible alternative to the current more complex reference standard technique. Adoption of a preload-free, single GBCA injection protocol will result in less GBCA usage and eliminate the possibility of quantitative errors due to variable dosing and incubation times (11). This could make it easier to have more widespread adoption of DSC-MRI using this simplified approach for patients with brain tumors. Furthermore, our results were robust whether nrCBV or srCBV techniques were used. This will also be advantageous in that it maintains Brain Tumor Imaging Protocol (BTIP) compliance where a single GBCA dose is required (25).

However, there are a number of study limitations that should be considered. First, the sample size was small and a number of studies were excluded during analysis. As noted above, there were a few outliers where the 30°/P-/C+ acquisition resulted in a higher rCBV than 60°/P+/C+ when the rCBV value was low. The reason for this is unclear and should be validated in a larger study, though our results overall showed strong correlation between the 2 methods in general. Second, data collection was retrospective and further validation in a larger sample size from multiple centers in a prospective fashion should be conducted. Third, only HGGs were studied and future studies should determine if our findings are true in other brain tumors. Fourth, our results were obtained on only three 1.5T scanners from the same manufacturer and software version. Future studies on a variety of 1.5T scanners from different manufacturers and software versions are needed. Fifth, our analysis only used the Boxerman-Schmainda-Weisskoff (BSW) model-based leakage correction method implemented in the analysis software from a single vendor. While the BSW method has been predicted to result in the highest accuracy and precision compared to other leakage correction methods such as γ-variate fitting (10, 20), variations in implementation could lead to differences in results. Sixth, the TE=45 ms recommended and used at 1.5T for both our 30°/P-/C+ and 60°/P+/C+ methods may result in lower contrast to noise ratios and be more susceptible to more EPI sequence artifacts. Future studies are needed to determine rCBV map interpretability and quality and their ability to predict clinical outcome (10, 11). Lastly, we were underpowered to determine if clinical factors such as glioma grade, IDH mutation status, MGMT promoter methylations status, number of recurrences and treatment history would affect rCBV correlations between the 2 methods but these questions could be addressed in a future larger study.

## Conclusion

In conclusion, 1.5T MRI scanners remain a significant field strength for brain imaging worldwide. Our results provide preliminary evidence that at 1.5T, a no-preload, low-FA acquisition method with model-based leakage correction could be a suitable alternative DSC-MRI method compared to the current reference standard intermediate-FA acquisition with full-dose preload and model-based leakage correction. If these results are validated in future prospective studies in a variety of brain tumors and scanners, then DSC-MRI acquisition at 1.5T will be more simplified and may encourage more widespread routine clinical use.

## Data availability statement

The raw data supporting the conclusions of this article will be made available by the authors, without undue reservation.

## Ethics statement

The studies involving human participants were reviewed and approved by Institutional Review Board, Keck School of Medicine of USC. Written informed consent for participation was not required for this study in accordance with the national legislation and the institutional requirements.

## Author contributions

MS, SYC contributed to conception and design of the study. SYC performed the statistical analysis. MS wrote the first draft of the manuscript. All authors contributed to the article and approved the submitted version.
